# The Influence of Full-Time Holistic Support Delivered by a Sports Nutritionist on Within-Day Macronutrient Distribution in New Zealand Provincial Academy Rugby Union Players

**DOI:** 10.3390/nu15010017

**Published:** 2022-12-21

**Authors:** Charlie J. Roberts, Nicholas D. Gill, Christopher M. Beaven, Logan R. Posthumus, Stacy T. Sims

**Affiliations:** 1 Faculty of Arts, Science and Technology, University of Northampton, Northampton NN1 5PH, UK; 2 Te Huataki Waiora School of Health, University of Waikato, Hamilton 3240, New Zealand; 3 New Zealand Rugby Union, Wellington 6011, New Zealand; 4 Sport Performance Research Institute New Zealand, Auckland University of Technology, Auckland 0627, New Zealand; 5 Faculty of Health, Education and Environment, Toi Ohomai Institute of Technology, Tauranga 3112, New Zealand

**Keywords:** rugby, meal timing, protein distribution, carbohydrate periodisation, sports nutritionist, behaviour change, nutrition support

## Abstract

Dietary intake is an important consideration for rugby union (‘rugby’) players to ensure substrate provision for optimal performance and facilitate recovery. Within-day meal distribution is especially important for athletes, particularly those with congested schedules and multiple daily training sessions. In the present study, 10 provincial academy rugby players engaged in a holistic support protocol informed by behaviour-change techniques led by a full-time sports nutritionist. Dietary intake was estimated during a 4-week monitoring and 4-week intervention period using the remote food photography method on one high-volume training day (two training sessions) and two low-volume training days (≤1 training session) per week. Lean body mass did not change significantly in response to the intervention. Significant increases were observed for protein on both low-volume (breakfast, AM snack, evening snack) and high-volume (post-gym, AM snack, evening snack) training days. Carbohydrate intake post-intervention was significantly greater at the pre-gym eating occasion but lower at PM snack and dinner eating occasions on high-volume days. These data suggest that incorporating a holistic support protocol led by a sports nutritionist can influence within-day nutrient intake in rugby players; however, no change to lean body mass was observed, and the influence of these changes in nutrient intake on performance and recovery warrants further investigation.

## 1. Introduction

Rugby union (‘rugby’) is a team sport played over 80 min with players engaging in high-intensity movement patterns interspersed with low-intensity bouts of walking and standing. Frequent collisions from tackles, scrums, rucks, and mauls are an integral part of rugby match-play [1]. Individual nutrient requirements between rugby athletes are expected due to differences in on-field roles and body composition requirements between positions [2]. Furthermore, a consideration for the distribution of food, meals, and beverages and thus energy and macronutrients throughout the day may play an important role in assisting the optimisation of performance and recovery in athletes [3].

Best-practice protein recommendations (1.2–2.0 g·kg·day^−1^) appear to be met or exceeded in professional and semi-professional rugby players [4,5,6,7,8,9,10,11]. Academy rugby players represent an important bridge between the amateur and semi-professional game and, whilst players in the UK appear to exceed recommendations [11], best-practice recommendations may not be consistently met in provincial academy players in New Zealand [12]. Additionally, muscle protein synthesis rates peak between 45 and 90 min following dietary amino acid ingestion, after which rates return to baseline by 180 min [13]. As such, consistent consumption of a proposed 0.4 g‧kg^−1^ of dietary protein across 4–6 evenly distributed meals has been suggested to facilitate a greater positive protein balance, skeletal muscle re-modelling, and possibly strength and performance; such responses may benefit a rugby athlete, as increased muscle strength directly relates to success in many rugby events, such as scrums, lineouts, tackles, and acceleration [14,15,16].

As rugby match-play is characterised by intermittent movement patterns interspersed with low-intensity periods over a prolonged period, promoting adequate carbohydrate intake in athletes to support training, performance, and recovery is a vital part of the sports nutrition practitioners’ role. Unlike dietary protein, best practice recommendations for daily carbohydrate intake (6–10 g·kg·day^−1^) are often not met in rugby players [17]. Ensuring carbohydrate intake is appropriate to maximise muscle and hepatic glycogen stores prior to training and competition, which can delay the onset of fatigue; furthermore, delaying carbohydrate consumption by two-hours following rugby match-play may impair muscle glycogen re-synthesis, which may negatively influence subsequent performance during training and matches [18,19]. This impairment may be of importance when athletes partake in congested schedules by balancing sport, training, study, and/or work commitments whilst maintaining social lives.

Sports nutritionists can implement interventions to facilitate positive dietary changes in athletes; however, limited practitioner support is often provided, particularly in academy settings [12,20]. As such, the aim of this study was to examine whether full-time holistic support informed by behaviour-change techniques provided by a sports nutritionist to athletes in a provincial rugby union academy in New Zealand influenced the within-day distribution of protein and carbohydrates. It was hypothesised that the incorporation of a nutrition support protocol delivered by a sports nutritionist would result in increased within-day protein and carbohydrate intake, subsequently increasing lean body mass.

## 2. Materials and Methods

### 2.1. Participants

Seventeen players from a sub-elite provincial rugby union academy volunteered to participate in the study. At the time of data collection, all participants trained with both the provincial academy and their local club alongside engaging full-time in outside obligations, such as work and study. Participants provided informed consent and ethical approval was obtained by the University of Waikato Human Research Ethics Committee (HREC(Health)2020#46).

### 2.2. Study Protocol

A non-randomised cross-over study design was implemented. All participants completed the monitoring period followed by the intervention period, with randomisation unfeasible due to the nature of the intervention. An initial four-week monitoring period allowed for the determination of habitual dietary intake followed by an intervention period. Dietary intake was recorded on four high-volume days (participants engaged in a morning and evening training session) and eight low-volume days (participants engaged in ≤1 training session) during the monitoring and intervention periods.

The nutrition support protocol was informed by behaviour-change techniques, with the techniques and method of implementation in the present study detailed elsewhere [12,21]. Support from a full-time sports nutritionist was provided to participants on both a group and individual level, with ongoing support provided during the intervention period. Participants attended a group seminar whereby overall group nutrient intakes were highlighted with information provided regarding meeting macronutrient requirements and improving diet quality, from both a health and athletic standpoint. Participants were provided with printed resources containing information detailing how to appropriately structure a core meal to meet nutrition requirements for an athlete. A range of nutritional recommendations were provided to account for the heterogeneity in body composition and on-field demands of the participants, which were then customised during individual consultations. Printed resources were also provided, namely recommended example meals, snacks, recipes for complex meals, and a grocery list.

Participants attended an initial 10 min consultation with the lead researcher to discuss personal goals and identify strategies to assist the individual reach overall and per-meal nutrition targets. Throughout the intervention period, regular in-person contact was maintained during training sessions and remote contact was maintained via an end-to-end encrypted cellular messaging device (WhatsApp, Inc., Santa Clara, CA, USA). A third-party batch-tested whey protein supplement was provided to each athlete at the beginning of the intervention period (Combat 100% Whey, MusclePharm, Calabasas, CA, USA batch #A163830620A).

### 2.3. Lean Body Mass

Lean body mass was measured using a three-dimensional optical scanning device (FIT3D^®^, San Mateo, CA, USA). Such scanners produce a three-dimensional avatar of the body allowing for anthropometric assessment [22]. The device was installed in a room with no natural light interference and shiny surfaces covered. Participants were instructed to create an account with Fit3D, allowing for repeated use and their own access to body composition results. Following account creation, users were presented with instructions via the attached monitor for accurate lean mass analysis. The participants were asked to remove all clothing down to their underwear and tie their hair up above their head if necessary. Prior to commencement of the scan, participants were required to hold two handles, allowing for an anatomical position to be maintained. Participants were asked to stand still and look forward for the 40 s scan duration. Lean body mass analyses were conducted at baseline, immediately prior to delivery of the nutrition support intervention, and in the final week of the intervention period. The coefficient of variation ranges for between-day reliability of the device used for lean mass and fat mass were 0.0–1.8% and 0.3–6.4%, respectively. FIT3D^®^ scanners have previously demonstrated equivalence to a 4-compartment model for lean mass measurements [22].

### 2.4. Dietary Analysis

Dietary intake was monitored using a remote food photography method using a mobile phone application (MealLogger, Wellness Foundry, Ashburn, VA, USA). MealLogger was chosen, as it allows users to upload photographs with descriptions to assist with the identification and analysis of foods or items. Additionally, the application is preferred to traditional dietary analysis methods, such as weighed food diaries, indicating greater compliance may be expected [23].

Participants were instructed to take a clear photo of all meals, food items, or beverages consumed, including a hand, pen, or cutlery in the picture to act as a size reference. Multiple pictures at different stages were encouraged if participants were preparing complex meals. Participants were encouraged to provide as much detail in the description box as possible to assist with the analysisdetailing other common household measures, including brand names and portion sizes; and details of cooking methods, condiments, or beverages. Additionally, participants were asked to use weighing scales if available.

Participants were contacted for any clarifications regarding logged items or meals if the quality of either photographs or descriptions was inadequate using WhatsApp. Additionally, each participant was contacted the morning following each monitoring day to both provide clarification on uploads if required and to recall the previous day’s intake and disclose unlogged items if appropriate.

Dietary intake was analysed using FoodWorks 10 (Version 10.0.4266, Xyris Software, Australia), as it contains a comprehensive database of food items in both Australia and New Zealand. If a food item was not present in FoodWorks, energy and macronutrient information was collected from food labels or the company website. Analysis was conducted by a single registered associate nutritionist with the Nutrition Society of New Zealand for consistency.

Per-meal absolute and relative to body mass protein and carbohydrate intake were calculated for the high-volume days and the combined low-volume/off days. This was to account for participants attending an academy training session at 05.30 on high-volume days, which may influence the consumption pattern of meals in the morning. Meals were separated into six eating occasions as described previously on low-volume days: breakfast, AM snack, lunch, PM snack, dinner, and evening snack [10,24,25]. On high-volume training days, meals were separated into seven eating occasions: pre-gym, post-gym, AM snack, lunch, PM snack, dinner, and evening snack.

### 2.5. Statistical Analysis

Statistical analyses were conducted using SPSS (Version 27, IBM corp., Armonk, NY, USA) with significance set at *p* ˂ 0.05. Shapiro–Wilk testing was applied to determine normality, with lean body mass being parametric and dietary intake data non-parametric. Descriptive statistics are displayed as means ± SD. A repeated-measures ANOVA with Greenhouse–Geisser correction was used to determine whether significant changes in lean body mass were observed between time-points. Dietary intake data were cleaned using the ‘replace missing values’ function to account for missing and inadequately logged days and allow for analysis. Distribution of nutrient intakes between meals were analysed using related-samples Friedman’s two-way analysis of variance by ranks, with Wilcoxon signed-rank sums test applied to compare changes between meals in response to the intervention.

## 3. Results

### 3.1. Participants

Two participants were excluded due to injury and illness, four were excluded due to inadequate adherence to the study procedures, and one was excluded due to leaving the academy. Dietary analysis was therefore conducted on 10 participants (age: 20.7 ± 1.7 years; body mass: 103.3 ± 18.8 kg; height: 186.8 ± 9.1 cm). Incomplete body composition data were collected for one participant included in the dietary analysis and as such, lean body mass data was analysed for nine participants. There was no significant change in lean body mass observed between time-points (baseline: 82.8 ± 13.0 kg; pre-intervention: 81.8 ± 12.8 kg; post-intervention: 82.2 ± 12.3 kg, *p* = 0.315).

### 3.2. Macronutrient Distribution

**Figure 1:**
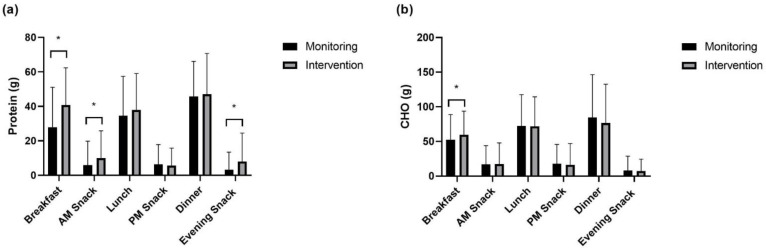
Absolute between-meal intake on low-volume training days of (**a**) protein and (**b**) carbohydrate. * signifies a difference in intake between monitoring and intervention periods.

**Figure 2:**
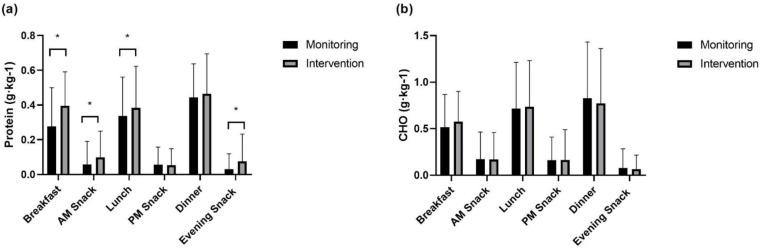
Relative between-meal intake on low-volume training days of (**a**) protein and (**b**) carbohydrate. * signifies a significant difference in intake between monitoring and intervention periods.

**Figure 3:**
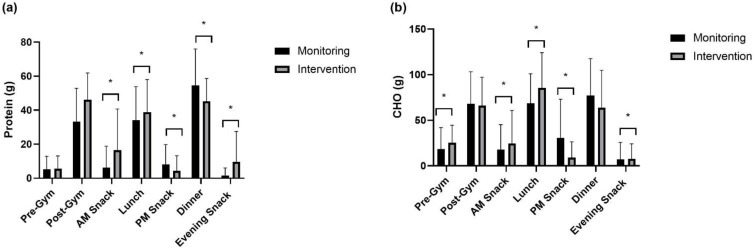
Absolute between-meal intake on high-volume training days of (**a**) protein and (**b**) carbohydrate. * signifies a significant difference in intake between monitoring and intervention periods.

**Figure 4:**
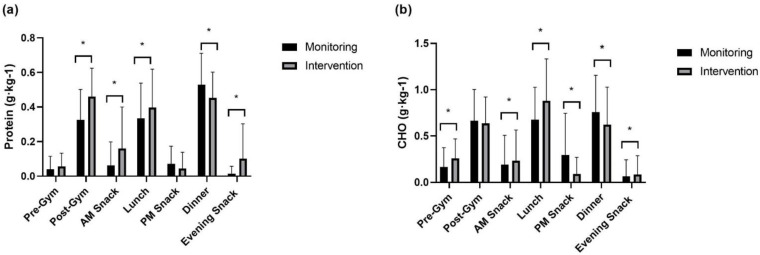
Relative between-meal intake on high-volume training days of (**a**) protein and (**b**) carbohydrate. * signifies a significant difference in intake between monitoring and intervention periods.

Absolute and relative protein and carbohydrate intakes are displayed in Figure 1, Figure 2, Figure 3 and Figure 4. On low-volume training days, both absolute and relative protein intake were significantly greater at ‘breakfast’ (absolute: *p* < 0.001, relative: *p* < 0.001), ‘AM snack’ (absolute: *p* = 0.001, relative: *p* < 0.001), and ‘evening snack’ (absolute: *p* < 0.001, relative: *p* < 0.001) in response to the intervention. Additionally, relative ‘lunch’ protein intake was significantly greater following the intervention (*p* = 0.044). A significant increase in absolute carbohydrate intake was observed at breakfast (*p* = 0.043), with no significant changes in between-meal relative carbohydrate intake.

On high-volume training days, both absolute and relative protein intake were significantly greater following intervention at ‘post-gym’ (absolute: *p* < 0.001, relative: *p* < 0.001), ‘AM snack’ (absolute: *p* < 0.001, relative: *p* < 0.001), and ‘evening snack’ (absolute: *p* < 0.001, relative: *p* < 0.001) time-points. Significantly greater relative protein intake was recorded at ‘lunch’ (*p* = 0.026), whilst a significant reduction in absolute (*p* = 0.008) and relative (*p* = 0.031) protein intake was observed at ‘dinner’ following the intervention. Absolute and relative carbohydrate intake were significantly greater at ‘pre-gym’ (absolute: *p* = 0.030, relative: *p* = 0.018), ‘AM snack’ (absolute: *p* = 0.028, relative: *p* = 0.048), ‘lunch’ (absolute: *p* < 0.001, relative: *p* < 0.001), and ‘evening snack’ (absolute: *p* = 0.042, relative: *p* = 0.047). Absolute (*p* < 0.001) and relative (*p* < 0.001) ‘PM snack’ carbohydrate intakes were significantly lower, and relative ‘dinner’ (*p* = 0.049) carbohydrate intake was significantly reduced during the intervention period.

## 4. Discussion

The purpose of this study was to examine the impact of full-time holistic support provided by a sports nutritionist to athletes in a provincial, sub-elite rugby union academy in New Zealand on the within-day distribution of protein and carbohydrates and subsequent changes to lean body mass. The implementation of full-time support may have facilitated a more balanced intake of dietary protein towards a recommended 0.4 g‧kg^−1^ per meal without a corresponding change in lean body mass [14,15]. Absolute carbohydrate intake was greater at breakfast on low-volume training days and differed at multiple eating occasions on high-volume training days.

Dietary protein intake was greater at multiple eating occasions on both low-volume and high-volume training days. The likely major influence on dietary protein intake was the incorporation of behaviour-change techniques into the intervention. Specifically, ‘adding objects to the environment’ in the form of supplemental dietary protein likely encouraged greater consumption at multiple eating occasions. Dietary protein intake significantly increased at the breakfast eating occasion on low-volume training days and the post-gym eating occasion on high-volume days (usually corresponding with breakfast due to the timing of morning training) towards the proposed 0.4 g‧kg^−1^ per meal threshold [14,15]. The distribution of adequately dosed protein throughout the day has been reported as a substantial factor in promoting a positive protein balance; therefore, it may be benefit the development and/or retention of lean mass [14,15,26,27]. As increased lean mass can amplify speed, strength, and power, rugby players may benefit from increased levels to support optimal match-play demands [16,28,29]. The results of the present study report no influence of manipulating per-meal dietary protein intake on lean mass development in rugby union players, with similar observations made by MacKenzie et al. [30]. Nonetheless, aiming for the proposed threshold at multiple eating occasions will assist athletes with increasing their total daily protein intake and whole-body protein balance, which may accumulate to chronic lean mass and metabolic adaptations beneficial to the rugby player [16].

Increased amino acid availability due to pre-sleep protein ingestion has been proposed as a strategy to amplify acute overnight muscle protein synthesis and thus chronic resistance training adaptations [31,32]. A greater intake at this time may better support positive muscle turnover, and 40 g of protein has been suggested to account for the greater post-absorptive period experienced during sleep than during a typical day [31]. Longitudinal studies in recreationally active males suggested pre-sleep protein intake can improve lean mass and strength; however, inconsistent endurance training adaptations were observed [32,33,34]. In the present study, both pre- and post-intervention protein intakes for both high-volume and low-volume training days were below 10 g. These data align with the sub-optimal protein intake previously observed at this feeding occasion among rugby, soccer, and mixed athletes [10,24,25].

Carbohydrates are a major fuel for rugby players due to the sport’s intermittent style of play, distance covered during matches, and high-intensity events, such as scrummaging and jumping [35]. In the present study, carbohydrate intake was measured both pre- and post-training around the morning sessions on a double training day. Following guidelines for carbohydrate intake, both pre- and post-training carbohydrate intake may have been inadequate to support optimal performance, adaptation, and recovery for the participants evening training session [17].

Immediate consumption of carbohydrates post-exercise results in greater muscle glycogen repletion than delayed feeding, the results of which have been demonstrated following rugby match-play [19,36]. As such, inadequate carbohydrate consumption following the morning training session on high-volume days may compromise the availability of substrates during the evening session, particularly when overall daily carbohydrate consumption is insufficient. Carbohydrate intake following training on high-volume days did not change in response to the intervention, and intakes may have been at the lower end of the values postulated to sufficiently replete muscle glycogen [3]. Despite specific pre-exercise carbohydrate recommendations being made for prolonged endurance exercise exceeding 90 min, muscle glycogen depletion has been observed in response to resistance training protocol, with the degree of depletion dependent on training intensity, duration, and work accomplished [17,37,38]. Additionally, the limited upregulation of cell signalling pathways that promote glycogen synthesis may be observed following resistance exercise, which may present a challenge for those with congested schedules and multiple training sessions in the day [37,39]. In the present study, morning resistance training sessions were succeeded by evening field-based training sessions consisting of intermittent movement patterns and skill-based activities, which may be impaired by carbohydrate insufficiency.

A reduction in carbohydrate intake at multiple eating occasions, as observed in the present study, may be counter-productive, particularly when athletes are not meeting best-practice sports nutrition recommendations, such as those in the current cohort [5,12,17]. Despite this, rugby players may experience similar degrees of glycogen depletion when consuming less carbohydrates than recommended in the literature in the days prior to rugby match-play [35]. As such, conclusions cannot be made regarding the impact of reduced carbohydrate intake in the present study without corresponding metabolic or performance data.

### Limitations and Directions for Future Research

Consideration for the limitations in the present study is warranted. Whilst reported nutrient intakes throughout the day were quantified, the influence of these changes on performance and recovery were not documented. Although best-practice recommendations from the literature are used for the basis of this manuscript for protein and carbohydrate intake at meals, it is unclear what impact these changes had in the current cohort.

The duration of intervention presents a limitation. Whilst the monitoring and intervention periods over 4 weeks are likely to be more representative of habitual dietary intake, this may not have been suitable to observe major shifts in dietary intake [40]. Indeed, whilst statistically significant changes were observed at numerous eating occasions for protein and carbohydrate, it is important to consider the ecological validity of these changes.

Future research exploring nutrient distribution should aim to incorporate match days. To reduce participant burden, this was not considered for the present study; however, the omission of this day is significant, as match days represent arguably the most important day for an athlete to consider for timing meals. For example, two hours of delayed carbohydrate feeding following rugby match-play has been demonstrated to lead to unclear muscle glycogen repletion, whilst immediate feeding very likely increases post-match muscle glycogen levels [19].

## 5. Conclusions

In conclusion, the incorporation of a holistic nutrition practitioner-supported protocol informed by behaviour-change techniques resulted in significant changes to protein and carbohydrate intake at multiple eating occasions in provincial academy rugby players; however, no significant changes to lean body mass were observed. Future research should explore the acute and chronic impacts of within-day macronutrient intake on performance and recovery to better inform rugby-specific overall and per-meal nutrient recommendations.

## Data Availability

The data presented in this study are available on request from the corresponding author.
